# Associated risk factors in the early stage of diabetic retinopathy

**DOI:** 10.1186/s40662-019-0148-z

**Published:** 2019-08-01

**Authors:** Fan Tan, Qi Chen, Xiran Zhuang, Chaoming Wu, Yanying Qian, Yuanyuan Wang, Jianhua Wang, Fan Lu, Meixiao Shen, Yingzi Li

**Affiliations:** 1 School of Ophthalmology and Optometry, Wenzhou Medical University, 270 Xueyuan Road, Wenzhou, Zhejiang China 325027; 2 Department of Ophthalmology, West China-Guang’an Hospital, Sichuan University, Guang’an, Sichuan China; 3 Department of Ophthalmology, Sichuan University West China Hospital, Chengdu, Sichuan China; 4 The Second Affiliated Hospital & Yuying Children’s Hospital of Wenzhou Medical University, Wenzhou, Zhejiang China; 5 Bascom Palmer Eye Institute, University of Miami Miller School of Medicine, Miami, FL USA; 6 Electrical and Computer Engineering, University of Miami, Miami, FL USA

**Keywords:** Diabetic retinopathy, Optical coherence tomography angiography, Retinal capillary density, Systematic risk factors

## Abstract

**Background:**

To investigate the retinal capillary density (RCD) of the macula using optical coherence tomography angiography (OCT-A) in type 2 diabetic patients and to further determine the association with risk factors.

**Methods:**

A total of 212 eyes from 212 subjects were recruited; subjects included diabetics with no retinopathy (NDR, *n* = 90 eyes), diabetics with mild retinopathy DR (MDR, *n* = 36 eyes), and healthy participants (Control, *n *= 86 eyes). All participants underwent OCT-A scanning. RCD was quantified by superficial and deep retinal capillary layers (SRCL and DRCL) from OCT-A images.

**Results:**

RCD in SRCL and DRCL was lower in NDR (*P* < 0.001) as well as in MDR (*P* < 0.001) when compared with control eyes. Diabetic patients were subdivided according to individual risk factors, complications related to diabetes, and hyperglycemia. Diabetic patients showed lower RCD in both the SRCL and DRCL when compared with healthy controls. Diabetics with age > 55y, HbA1c > 7% had significantly reduced DRCL (*P* < 0.05) when compared with the other group of diabetics (age < 55y, HbA1c < 7%). Diabetics with a blood urea nitrogen (BUN) > 8.2 mmol/L had significantly reduced SRCL and DRCL when compared to the other group of diabetics.

**Conclusions:**

Risk factors including older age, higher level of HbA1c, LDL-C and BUN, were associated with lower RCDs found in type 2 diabetic patients with and without mild DR by OCT-A. The impairment of retinal capillary by OCT-A may play a key role in the early monitoring of management in diabetes.

## Background

Diabetes mellitus (DM) is characterized by chronic hyperglycemia [1] and the development of diabetes-specific macrovascular and microvascular alterations [2, 3]. Diabetic retinopathy (DR), one of the common microvascular complications relating to diabetes, is a leading cause of vision impairment and vision loss among adults [1, 4]. Although the causes of vascular damage as well as visual function abnormality are not fully understood, evidence from histopathologic studies has shown that the changes in the retinal capillaries precede the first clinical visible retinal signs, such as microaneurysms [5–9]. Therefore, early surrogate clinical biomarkers that can detect and quantify the preclinical lesions of retinal capillaries in diabetic patients are needed to predict the development of DR from the earliest stages and allow for early intervention to eventually retard or prevent retinopathy.

Fluorescein angiography (FA) [10, 11], or color fundus photographs [3, 12–14], have been widely used to establish the categorical grading of non-perfusion in DR. However, grading with FA is seldom done in the routine clinical practice due to its invasive nature. In addition, these two modalities do not evaluate the retinal capillary network reliably and cannot detect subtle changes due to their low resolution [15–17]. Optical coherence tomography angiography (OCT-A) is a novel imaging modality that can noninvasively and quickly demonstrate the intra-retinal microvasculature with quantitative and objective assessments [18–21]. Various parameters such as macular vascular density [22–24], macular vascular fractal dimension [22–24] and the area of foveal avascular zone (FAZ) [22–24], have been used to quantify retinal perfusion in diabetic patients with or without clinical DR by using OCT-A. In fact, type 2 DM is a disease of middle-aged and elderly individuals who have a higher prevalence of hypertension and are at higher risk of cardiovascular disease and mortality. Therefore, apart from the conventional DR risk factors (e.g., duration of DM or glycated hemoglobin A_1C_ [HbA_1c_]) [25], it is also important to examine the effects of other systemic risk factors (e.g., age, sex, body mass index [BMI], blood pressure as well as relevant venous blood test parameters) on the retinal microvasculature in patients with type 2 DM, which are still unclear currently. The purpose of this study is to quantify the retinal capillary density (RCD) of the macula using OCT-A in type 2 diabetic patients with or without mild retinopathy, and to further evaluate the associations with risk factors.

## Methods

### Study population

Healthy volunteers and type 2 DM participants were recruited from the endocrine department of the Second Affiliated Hospital & Yuying Children’s Hospital and the Fundus Clinic of the Eye Hospital, Wenzhou Medical University, Wenzhou, China, from November 2015 to October 2017. Participants with high myopia, nondiabetic macular pathology, media opacity, or other significant eye diseases were excluded. Informed consent was obtained, and the study was approved by the ethics committee of the Eye Hospital of Wenzhou Medical University and complied with the tenets of the Declaration of Helsinki. All diabetic patients were initially graded into two subgroups according to the International Clinical Diabetic Retinopathy Severity Scale [26]: DM without retinopathy (NDR), and mild non-proliferative DR (MDR).

Demographic information collected from the patients included age, sex, duration of DM, smoke and alcohol history as well as their treatment history for DM. In addition, BMI, systolic blood pressure (SBP), diastolic blood pressure (DBP), venous blood tests, and ophthalmologic examinations were performed on each patient. BMI was calculated as weight in kilograms divided by the square of height in meters. Venous blood was obtained for measurement of HbA_1C_, serum triglyceride (TG), high-density lipoprotein cholesterol (HDL-C), low-density lipoprotein cholesterol (LDL-C), total cholesterol (TC), blood urea nitrogen (BUN), and serum creatinine. Ophthalmologic examinations included assessment of best-corrected visual acuity (BCVA) using a logarithm of the mild angle of resolution (LogMAR), intraocular pressure (IOP) measurement, slit-lamp biomicroscopy, axial length (AL) measurement and three ophthalmoscopy methods, including slit-lamp biomicroscopy with a fundus pre-set lens, color fundus photographs (KOWA nonmyd α-DIII, 8300, Japan) and panoramic wide-angle laser fundus camera (ophthalmoscope-Daytona, P200T, Optos, UK). One masked specialist (LY) used the three ophthalmoscopy methods to confirm the subgroups (NDR or MDR). Healthy controls were recruited during the same study period from subjects who received annual eye examinations or from the family members of the patients.

### OCT-A measurements and segmentation

All the enrolled subjects were imaged by an OCT-A system (Optovue, RTVueXR Avanti; Optovue, Inc., Fremont, CA, USA). The Optovue takes 304 × 304 volumetric A-scans at 70,000 A-scans per second, which uses the proprietary split-spectrum amplitude-decorrelation angiography (SSADA) algorithm to produce detailed images and to minimize scan acquisition time. Dense horizontal and vertical raster cubes are acquired and combined by a proprietary algorithm to reduce motion artifacts. All subjects used Motion Correction Technology to reduce motion artifact for ultra-high resolution images for the 3 × 3 mm image acquisition area centered on the fovea. The OCT instrument automatically detected and separated the retina vascular layer into the superficial and deep retinal capillary plexuses (SRCL, DRCL, respectively). The superficial retinal capillary layer extended from 3 μm below the internal limiting membrane to 15 μm below the inner plexiform layer (IPL). The deep retinal capillary layer extended from 15 to 70 μm below the IPL, which included both the intermediate and deep capillary plexus. If there was an obvious deviation for the segmentation obtained by the OCT-A system, the segmentation lines were then adjusted manually.

RCD (%) was defined as the proportion of the measured area occupied by perfused vessels, which was defined as pixels having signal acquired by the SSADA algorithm. It was used to characterize the vascular structural information and was calculated on the imported raw OCT-A images in PNG format by our custom automated algorithm, as described in our previous study [27]. The grayscale of each two-dimensional OCT-A image was first extended by bicubic interpolation to 1024 × 1024 pixels to enhance image details. Next, a two-way combined method consisting of a canny edge detector algorithm and a level set algorithm was used to detect the boundary of FAZ. The area within the FAZ, having a circle of fixed radius (diameter = 0.6 mm), was then determined to establish the baseline signal to-noise ratio for global thresholding. This image was then separately processed to generate two binary images by global thresholding and adaptive thresholding: the first image contained only the large blood vessels. The other binary image contained large and small vessels. The two resulting binary vessel maps were subtracted to obtain a binary image containing only the small vessels. RCD was then calculated for the 2.5 mm diameter zone after excluding the FAZ (diameter = 0.6 mm). All the above methods were performed using MATLAB v.2015a (Mathworks Inc., Natick, MA, USA).

### Statistical analysis

All data were expressed as the mean ± standard deviation. One-way analysis of variance (ANOVA) was used to test for differences among groups. Chi-squared test was used to test for differences in sex, eye laterality, smoking history, alcohol and insulin therapy. The diabetic group, including NDR and MDR, were divided into two new-subgroups, according to each individual characteristic, hyperglycemia, and complications related to diabetes, respectively. The relationships between the RCD and systemic factors were evaluated by the differences between each newly divided subgroup. All data were analyzed with SPSS software (version 23.0; SPSS Inc., Chicago, Illinois, USA). *P* values < 0.05 were considered statistically significant.

## Results

A total of 212 eyes from 212 subjects were recruited, including type 2 DM (126 eyes) and 86 healthy participants (86 eyes). Segmentation lines were adjusted manually for 20 images from 10 eyes due to their obvious deviation errors. From the diabetic eyes, 90 eyes had NDR and 36 had MDR, respectively. There were no statistically significant differences in the demographic characteristics among the three groups, except for the BCVA (*P* < 0.001) and SBP (*P* = 0.031) (Table 1).

**Table 1:** Participant characteristics

	Control	NDR	MDR	*P*
	*n* = 86	*n* = 90	*n* = 36	
Eye (OD/OS)	47/39	52/38	21/15	0.892
Age (years)	52.5 ± 9.11	52.7 ± 11.91	56.61 ± 12.25	0.135
Sex (male/female)	31/55	46/44	16/20	0.131
BMI (kg/m^2^)	23.37 ± 2.84	23.86 ± 2.95	23.9 ± 3.04	0.613
SBP (mmHg)	123.48 ± 13.77	128.4 ± 16.22	132.31 ± 18.23	**0.031**
DBP (mmHg)	76.88 ± 9.44	79.26 ± 10.44	78.36 ± 9.35	0.373
MAP (mmHg)	118.12 ± 13.34	122.06 ± 14.32	122.46 ± 14.01	0.201
SE (Diopter)	0.15 ± 1.32	−0.18 ± 1.59	0.19 ± 1.48	0.262
BCVA (LogMAR)	0.98 ± 0.14	0.89 ± 0.15	0.86 ± 0.14	**< 0.001**
AL (mm)	23.24 ± 0.86	23.37 ± 0.94	23.12 ± 0.73	0.320
IOP (mmHg)	13.96 ± 2.65	14.8 ± 2.58	14.88 ± 2.95	0.117
Smoke (Y/N)	–	75/15	25/11	0.082
Alcohol (Y/N)	–	74/16	28/8	0.566
Insulin (Y/N)	–	74/16	24/12	0.058
Duration (years)	–	5.47 ± 5.34	7.02 ± 5.65	0.150
BG (mmol/L)	–	7.85 ± 3.93	8.09 ± 2.38	0.738
HbA1c (%)	–	7.55 ± 1.78	8.17 ± 1.98	0.114
BUN (mmol/L)	–	6.24 ± 5.15	6.04 ± 4.27	0.856
Creatinine (μmol/L)	–	60.62 ± 16.88	73.17 ± 91.96	0.272
TG (mmol/L)	–	1.99 ± 1.73	1.59 ± 1	0.229
TC (mmol/L)	–	4.54 ± 1	4.58 ± 0.96	0.859
HDL-C (mmol/L)	–	1.3 ± 0.37	1.2 ± 0.28	0.194
LDL-C (mmol/L)	–	2.48 ± 0.77	2.45 ± 0.98	0.866

Values are mean ± standard deviation for all subjects in each group. *Control* = control eyes; *NDR* = diabetic patients with no diabetic retinopathy; *MDR* = diabetic patients with mild diabetic retinopathy; *BMI* = body mass index; *SBP* = systolic blood pressure; *DBP* = diastolic blood pressure; *MAP* = mean arterial pressure; *SE* = spherical equivalent; *BCVA* = best corrected visual acuity; *AL* = axial length; *IOP*= intraocular pressure; *BG* = blood glucose; *HbA1c*= glycosylated hemoglobin; *BUN* = blood urea nitrogen; *TG* = triglyceride; *TC* = total cholesterol; *HDL-C* = high-density lipoprotein cholesterol; *LDL-C* = low-density lipoprotein cholesterol. *P*-values for differences among the three groups were determined by one-way ANOVA (Eye and Sex by Chi-Squared test) and difference between two groups by independent samples t-test. Bold *P*-value represents < 0.05

**Fig. 1:**
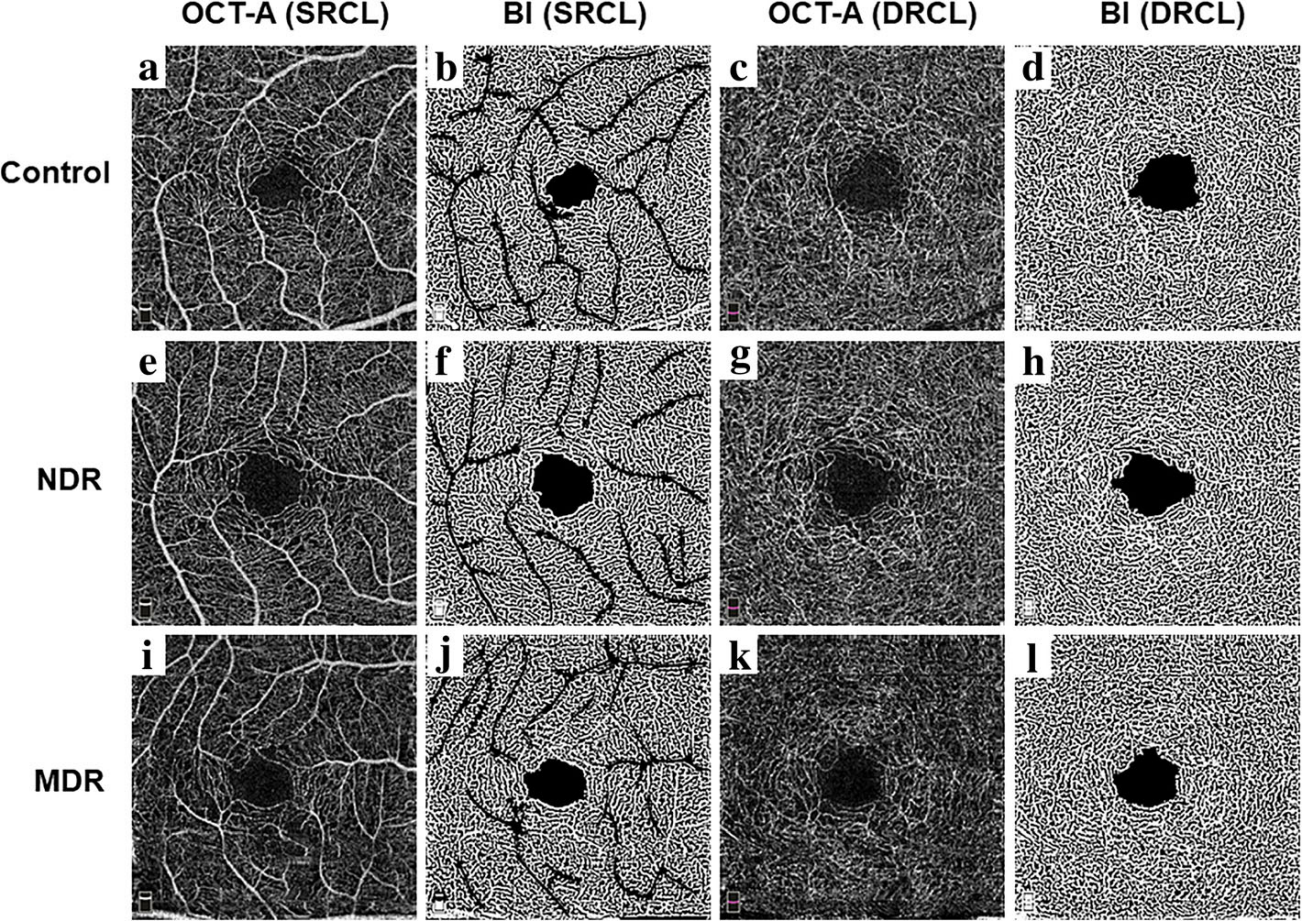
Representative optical coherence tomography angiography (OCT-A) and postprocessed images for a control, diabetes without retinopathy (NDR) and diabetes with mild retinopathy (MDR) eyes illustrating the automated analysis algorithm. Original OCT-A images of the superficial retinal capillary layers of control (**a**), NDR (**e**) and MDR (**i**) eyes and binary images of the capillary plexus after removing background noise and larger vessels of control (**b**), NDR (**f**) and MDR (**j**) eyes. Original OCT-A images of the deep retinal capillary layers of control (**c**), NDR (**g**) and MDR (**k**) eyes and Binary images of the capillary plexus after removing background noise of control (**d**), NDR (**h**) and MDR (**l**) eyes. Control, control eyes; NDR, diabetic patients with no DR; MDR, diabetic patients with mild DR; SRCL, superficial retinal capillary layer; DRCL, deep retinal capillary layer; BI: binary images

Raw OCT-A and their corresponding postprocessed binary images of the superficial and deep retinal capillary layers in the control eyes, and in the eyes of diabetic patients with NDR and MDR were acquired (Fig. 1).

### Retinal capillary density in three groups

**Fig. 2:**
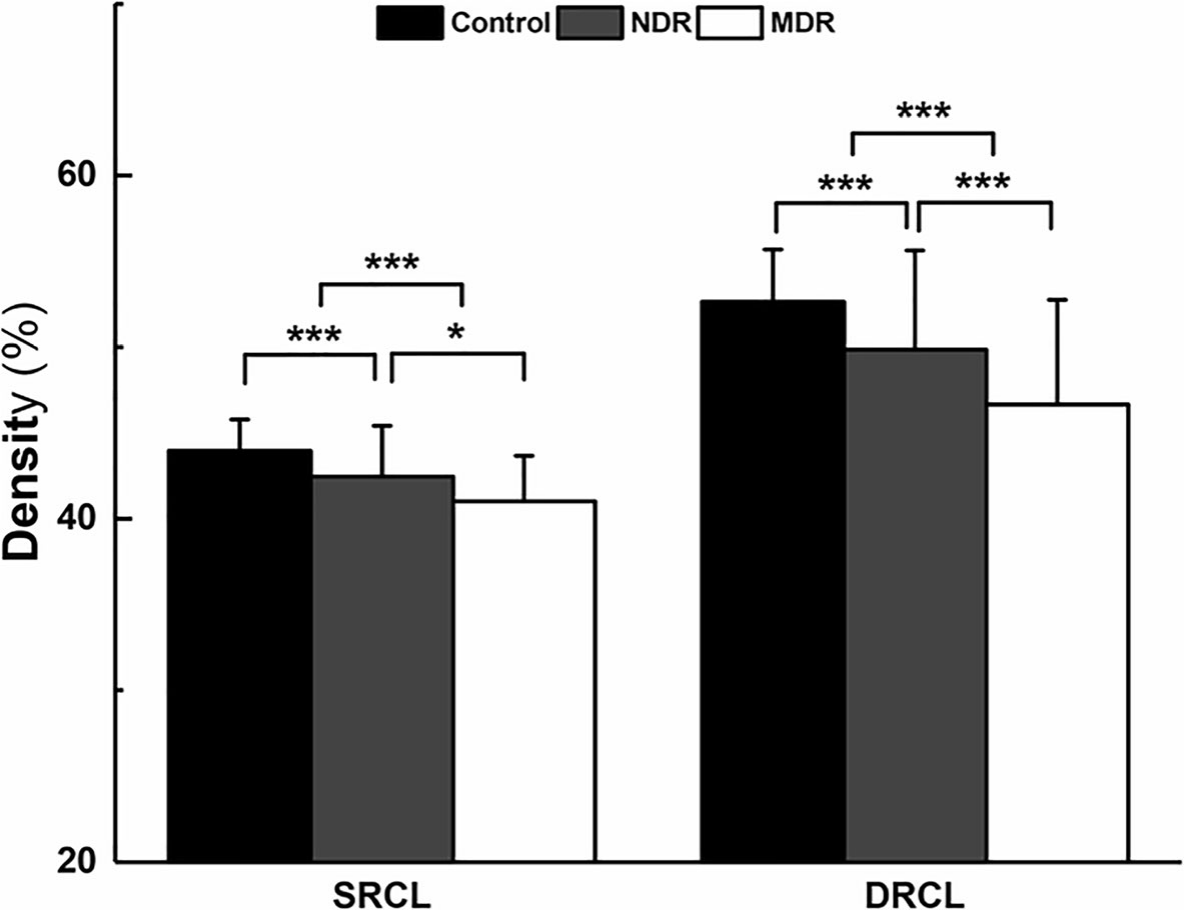
Comparisons of the retinal capillary density (RCD) in diabetic patients with controls on OCT-A images in the superficial and deep retinal capillary layers (SRCL and DRCL). The RCDs in the SRCL (**a**) and DRCL (**b**) of both the no DR (NDR) group and the mild DR (MDR) group were lower than those of the control group. In addition, the RCDs in the MDR group were also decreased compared to those of the NDR group. *P < 0.05, **P < 0.01, ***P < 0.001

As shown in Table 2 and Fig. 2, there were significant differences of the RCDs both in SRCL and DRCL among the three groups. In SRCL, the mean RCD was significantly lower in diabetic patients with MDR (41.0 ± 2.7%) compared with the NDR (42.4 ± 3.0%, *P* = 0.004, Table 2, Fig. 2) and healthy groups (43.9 ± 1.8%, *P* < 0.001, Table 2, Fig. 2). Moreover, there was also a statistically significant decrease of RCD in the NDR group compared with healthy participants (*P* < 0.001, Table 2, Fig. 2). In DRCL, the mean RCD in patients with MDR (46.6 ± 6.1%) was also significantly deceased compared with the NDR (49.9 ± 5.8%, *P =* 0.001) and healthy groups (52.6 ± 3.0%, *P* < 0.001 Table 2, Fig. 2). There was also a statistically significant decrease in RCD in the NDR group compared with the healthy groups (*P* < 0.001, Table 2, Fig. 2).

**Table 2:** RCD (%) in controls and diabetics with no or mild retinopathy

	Control	NDR	MDR	P (Control-NDR)	P(Control-MDR)	P (NDR-MDR)
SRCL	43.9 ± 1.8	42.4 ± 3.0	41.0 ± 2.7	**< 0.001**	**< 0.001**	**0.004**
DRCL	52.6 ± 3.0	49.9 ± 5.8	46.6 ± 6.1	**< 0.001**	**< 0.001**	**0.001**

*RCD* = retinal capillary density; *Control* = control eyes; *NDR* = diabetic patients with no diabetic retinopathy; *MDR* = diabetic patients with mild diabetic retinopathy; *SRCL* = superficial retinal capillary layer; *DRCL* = deep retinal capillary layer

*P* value for the comparisons between the three groups by ANOVA

Bold *P*-value represents < 0.05

### Influence of individual characteristics

**Fig. 3:**
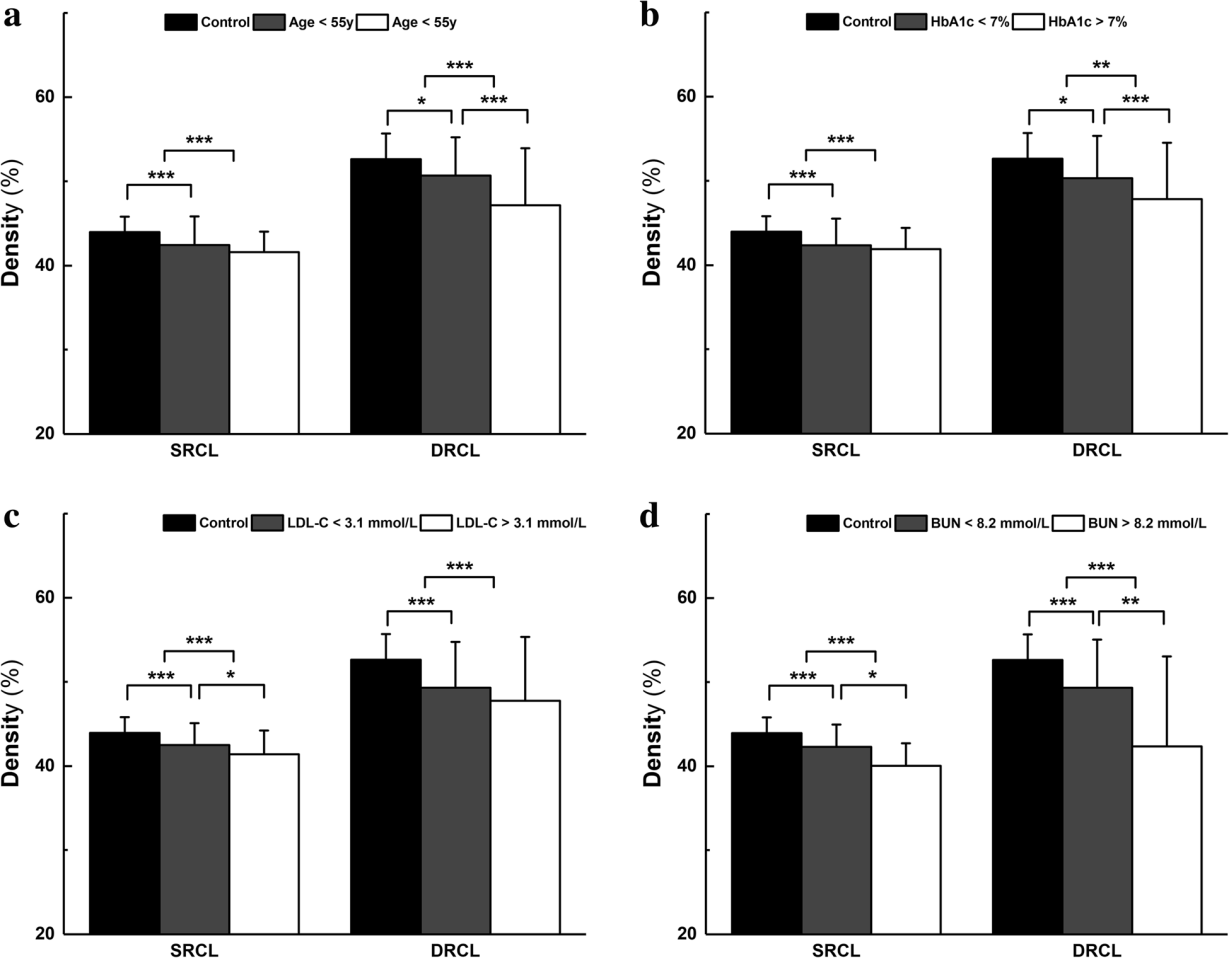
Comparisons of the retinal capillary density (RCD) in diabetic subgroups grouped by major risk factors with controls on OCT-A images in the superficial and deep retinal capillary layers (SRCL and DRCL). The RCDs in SRCL and the DRCL of diabetic subgroups grouped by major risk factors were lower than those of the control group. In addition, the RCDs in the diabetic groups with age > 55y (**a**), HbA1c > 7% (**b**), LDL-C > 3.1 mmol/L (**c**), or BUN > 8.2 mmol/L (**d**) were also decreased compared to those of the other groups. *P < 0.05, **P < 0.01, ***P < 0.001

The diabetic group, including NDR and MDR, was divided into two new-subgroups, according to each individual characteristic, such as age (<55y or > 55y), sex (male or female), BMI (< 25 kg/m^2^ or > 25 kg/m^2^), history of smoking and alcohol consumption (yes or no, respectively). In SRCL, when comparing the two newly formed diabetic subgroups with healthy controls, the RCDs of each diabetic subgroup were significantly deceased (all *P* < 0.01). However, the RCD in SRCL was not significantly decreased between the two diabetic subgroups (*P* > 0.05). In DRCL, the RCDs of each diabetic subgroup were also significantly decreased compared to those in the control group (all *P* < 0.05). In addition, when comparing each diabetic subgroup, the RCDs were significantly decreased in those with age > 55y compared with those with age < 55y in the DRCL (*P* < 0.001, Table 3, Fig. 3). No significant difference was seen between the diabetic subgroups when other individual characteristics were evaluated (*P* > 0.05).

**Table 3:** RCD (%) in diabetic patients grouped by major risk factors

	Percentage		Age	SRCL	DRCL
	NDR	MDR			
Control				43.9 ± 1.8	52.6 ± 3.0
Diabetes					
Age < 55y	81.5% (53)	18.5% (12)	–	42.4 ± 3.4^c^	50.7 ± 4.6^a^
Age > 55y	60.7% (37)	39.3% (24)	–	41.6 ± 2.4^c^	47.1 ± 6.8^c^
*P* Value				0.064	**< 0.001**
HbA1c < 7%	82.0% (41)	18.0% (9)	53.0 ± 12.5	42.4 ± 3.1^c^	50.3 ± 5.0^a^
HbA1c > 7%	63.9% (49)	36.1% (2)	55.8 ± 11.8	41.9 ± 2.5^c^	47.8 ± 6.7^c^
*P* Value			0.233	0.335	**0.010**
LDL-C < 3.1 mmol/L	71.6% (58)	28.4% (23)	55.1 ± 12.3	42.5 ± 2.6^c^	49.3 ± 5.5^c^
LDL-C > 3.1 mmol/L	68.0% (17)	32.0% (8)	51.2 ± 13.3	41.4 ± 2.8^c^	47.7 ± 7.6^c^
*P* Value			0.179	**0.035**	0.168
BUN < 8.2 mmol/L	71.0% (66)	29.0% (27)	54.4 ± 11.7	42.3 ± 2.6^c^	49.3 ± 5.8^c^
BUN > 8.2 mmol/L	60.0% (3)	40.0% (2)	53.2 ± 18.8	40.1 ± 2.7^c^	42.4 ± 10.7^c^
*P* Value			0.833	**0.033**	**0.002**

*RCD* = retinal capillary density; *Control* = control eyes; *NDR* = diabetic patients with no DR; *MDR* = diabetic patients with mild DR; *SRCL* = superficial retinal capillary layer; *DRCL* = deep retinal capillary layer; *HbA1c* = glycosylated hemoglobin A1c; *LDL-C* = low-density lipoprotein cholesterol; *BUN* = blood urea nitrogen

One-way ANOVA *P* values between diabetic groups and control group: a represents < 0.05; b represents < 0.01; c represents < 0.001. One-way ANOVA *P* values among subgroups of diabetes patients: bold *P*-value represents < 0.05

### Influence of hyperglycemia

The diabetic group, including NDR and MDR, was further divided according to hyperglycemia, which included the duration of diabetes (< 5y or > 5y), insulin treatment (yes or no) and HbA1c level (< 7% or > 7%). In SRCL, when comparing two new diabetic subgroups with controls, there was a significantly reduced RCD in two new diabetic subgroups in all sectors of SRCL (all *P* < 0.05). There was no significant difference (*P* > 0.05) when both subgroups of the diabetics were compared. In DRCL, the two new diabetic subgroups had lower RCD in DRCL compared with the controls (*P* < 0.05). Moreover, when the two diabetic groups were compared, the RCDs in DRCL in the diabetic subgroup with HbA1c > 7% were lower than those with HbA1c < 7% (*P* < 0.05, Table 3, Fig. 3).

### Influence of complications related to diabetes

In addition, the diabetic group was also divided according to various characteristics of complications related to diabetes, which included hypertension (SBP < 140 mmHg or > 140 mmHg, and DBP < 90 mmHg or > 90 mmHg), hyperlipidemia (TG < 1.7 mmol/L or > 1.7 mmol/L, TC < 5.2 mmol/L or > 5.2 mmol/L, HDL-C < 1.96 mmol/L or > 1.96 mmol/L, and LDL-C < 3.1 mmol/L or > 3.1 mmol/L) and renal dysfunction (BUN < 8.2 mmol/L or > 8.2 mmol/L, and Creatinine < 70 μmol/L or > 70 μmol/L). The SRCL showed significantly reduced (*P* < 0.05) RCD in each diabetic subgroup when compared to the controls with SBP, DBP, TG, TC, LDL-C, BUN and creatinine used as the parameters. When compared between each diabetic subgroup, the RCDs in SRCL in the diabetic subgroup with LDL-C > 3.1 mmol/L and BUN > 8.2 mmol/L were lower than those with LDL-C < 3.1 mmol/L and BUN < 8.2 mmol/L, respectively (*P* < 0.05, Table 3, Fig. 3). In DRCL, the RCDs of each diabetic subgroup were significantly reduced (*P* < 0.05) when compared to the healthy controls with SBP, DBP, TG, TC, LDL-C, BUN and creatinine evaluated as parameters. Furthermore, when compared between each diabetic subgroup according to various characteristics of complications related to diabetes, the RCDs in DRCL in the subgroup with BUN > 8.2 mmol/L were also lower than the other subgroup (*P* < 0.05, Table 3, Fig. 3).

We also determined whether diabetic retinopathy was correlated with RCD when adjusted for other factors. For all diabetes subjects (included NDR and MDR), multiple linear regressions showed that RCD in SRCL (*P* = 0.027) and DRCL (*P* < 0.001) was independently and negatively correlated with age after adjustment for other risk factors.

## Discussion

In this cross-sectional study, we demonstrated retinal capillary loss in type 2 diabetic patients with and without mild retinopathy by quantifying RCD using OCT-A. Additionally, we found that type 2 diabetic patients who were older, had higher HAb1c, LDL-C and BUN, had more severe capillary abnormalities. The ability of the indicator to quantify the impairment of retinal capillary network in diabetic patients especially in patients without clinical DR, has important implications for the early detection and prevention of retinopathy, and the understanding of the pathophysiological mechanisms underlying DR development [5, 6, 28]. The present study demonstrated that the RCD in diabetic patients without clinical DR was decreased compared with non-diabetic eyes, which was consistent with our previous study using the FD value [27]. However, Nesper et al. [29] reported that there was no significant difference in retinal vessel densities between the NDR patients and controls. One possible reason for such discrepancy might be whether the large vessels in the OCT-A images were removed in the analysis of retinal vessel density. In our study, the custom automated algorithm helped in the extraction of large vessels and the density of the capillaries were measured, which were also used in the previous study [30]. To the best of our knowledge, this is the first study to investigate the relationships between this early indicator and various diabetic risk factors, where we found that decreased RCD had a negative correlation with increasing age, and those with higher HAb1c, LDL-C and BUN.

Although studies have reported that intense glycemic control can prevent and delay the initiation of diabetic retinopathy [31, 32], the close association between diabetes and the prevalence of microvascular complications was also demonstrated [25, 31, 33, 34]. Some other studies found a substantial incidence of retinopathy, even among patients with good glycemic control [35]. Previous studies have shown that both increased FAZ and decreased vessel density were associated with worsening DR severity, including NDR/mild/moderate/severe non-proliferative DR and proliferative DR, using OCT-A [22–24, 29, 36]. Our study also showed decreased RCD in diabetic patients with mild DR compared with patients without retinopathy and controls. The decreased RCD in the early stages, reflecting ischemia of the macula, might be associated with the initiation of impaired retinal microvasculature in diabetic patients. Therefore, our results support the hypothesis that the RCD of the macula quantified by OCT-A may be a sensitive indicator of early retinal microvascular damage in diabetes even before the clinical signs of diabetic retinopathy occur.

Type 2 diabetic patients have a significant risk of blindness from diabetic retinopathy and are at higher risk of developing cardiovascular disease and mortality related to systematic microvascular complications [1, 2, 37, 38]. The current study investigated the associations between the early impairment of the retinal microvasculature and the risk factors of patients with type 2 DM, which might have a potential value for guiding clinicians. We found that only age, HbA1c, LDL-C and BUN levels have an obvious and reliable influence on the decreased RCD, especially in the DRCL, after we divided the diabetic patients into two subgroups by various risk factors. Wei et al. [39] investigated the age-related alterations in retinal microvascular network in healthy subjects using OCT-A and found that the retinal vessel density decreased during aging. The results were similar with those obtained in our study, even though the subjects of the current study were diabetic patients and the other risk factors of which might have some extra effect on the impairment of retinal microvasculature. Therefore, a huge database of age-matched healthy subjects as a reference is important in the clinical diagnosis of the microvascular damage of diabetic patients. As shown in our study which enrolled age-matched control subjects, the decreased RCD found in diabetic patients with and without MDR may represent a novel and specific biomarker for evaluating the early presence of microvascular damage related to type 2 DM. Dieren et al. [32] indicated that effective glycemic control was related to decreasing HbA1c levels. Cheung et al. [1] reported that a 1% decrease in glycated HbA1c might be roughly equal to a 40% decrease risk of retinopathy. In the present study, we also found that the HbA1c level of more than 7%, reflecting poor long-term blood sugar control, also had a major influence on the impairment of retinal microvessels in diabetic patients. In addition, we found that increasing LDL-C was another significant risk factor for decreasing the RCD and might aggravate the damage of retinal microvasculature in type 2 diabetic patients, which is consistent with previous studies. Papavasileiou et al. [40] reported that higher total and LDL cholesterol were associated with the presence of hard exudates, and a greater hard exudate area was measured in the fundus photographs in African Americans with type 2 DM. Ting et al. [24] found that hyperlipidemia was also associated with a reduced capillary density index using a swept-light source OCT-A device. Ivers et al. [41] demonstrated that diabetics with longer duration, and insulin treatment had a significantly increased risk for DR, however, this was not detected in the early stage of diabetic patients of our study. Therefore, diabetic patients with different severities of retinopathy in the two studies might be one of the main reasons for the discrepancy.

In our study, the more significant decreases of RCD in diabetic subgroups were found to occur in the DRCL compared to those in the SRCL. This may be attributed to the different characteristics of anatomical structures in the two layers. In fact, the DRCL has more abundant smaller vessels than the SRCL [42, 43]. In our previous study, we found that fractal dimensions had larger areas under the ROC curve in the DRCL than the SRCL, which indicated a higher diagnostic value [27]. Perhaps capillary vasoconstriction in the deep retinal capillary layer occurs earlier or more frequently to compensate for the reduced blood flow and the resulting hypoxia and ischemia in early DM, which has been reported in previous studies [27, 43, 44]. Moreover, in the present study, we found that the diabetic patients who were older, or with higher HbA1c, TG, LDL-C and BUN level had decreased RCD in the retina, especially in the DRCL. These might demonstrate that protection and treatment of the microvasculature in the DRCL in the early stage of DM are of a particular value.

Our study, however, has some limitations. First, the cross-sectional and non-interventional natures of the study limits our ability to determine the role of OCT-A in the prediction of incidence and progression of DR. A longitudinal study with a larger sample size is needed to further investigate the clinical utility of retinal capillary measurements based on OCT-A images as a potential tool to evaluate the early impairment of diabetic microvasculature in the future. Secondly, a venous blood test was not performed in the control subjects. Therefore, it cannot be excluded that some of the controls had undetected Type 2 DM or other systematic diseases. However, this may not impact our results because the non-diagnosed controls would most likely have led to an underestimate of the difference in RCD between the diabetic patients and the controls rather than an overestimate. In addition, although the OCT-A provided improved visualization of the superficial and deep capillary networks compared to FA, the current technology of OCT-A is limited by a small field of view (3 × 3 mm^2^). This may limit our understanding of the vascular changes in the peripheral retina in the early stages of DR. Finally, the present study showed that RCD in SRCL (*P* = 0.027) and DRCL (*P* < 0.001) was independently and negatively correlated with age after adjusting for other risk factors by multiple linear regression. The result showed it was indispensable that the interaction of age with other risk factors should to be considered in the future study. The lack of correlations among the other risk factors and RCD may be due to the small sample sizes, and thus further studies with larger populations are warranted.

## Conclusions

In summary, this study demonstrated, using OCT-A, that lower RCD (equating to more impaired vessels) were found in diabetic patients with and without mild DR. Aging, higher HbA1c, LDL-C and BUN level were found to be the major risk factors in the retinal microvascular impairment of type 2 diabetic patients, and OCT-A is considered to be a promising approach to detect early microvasculature impairment in diabetic patients by noninvasively evaluating capillary perfusion.

## Data Availability

All relevant data are within the paper and its supporting information files.
